# Editorial: Modeling brain function at the level of neurons and circuits *via* computational and data-driven approaches

**DOI:** 10.3389/fncom.2022.965735

**Published:** 2022-08-15

**Authors:** Ranjit Kumar Upadhyay, Dibakar Ghosh, M. A. Aziz-Alaoui, Muhammet Uzuntarla

**Affiliations:** ^1^Department of Mathematic and Computing, Indian Institute of Technology (Indian School of Mines), Dhanbad, India; ^2^Physics and Applied Mathematics Unit, Indian Statistical Institute, Kolkata, India; ^3^Normandie University, UNIHAVRE, LMAH FR-CNRS-3335, ISCN, Le Havre, France; ^4^Department of Bioengineering, Gebze Technical University, Darica, Turkey

**Keywords:** dynamical systems, complex networks, neuroscience, chaos, information processing, Glial cells, astrocytes, artificial intelligence

The issue of modeling brain function that corresponds to the interaction between neurons in the form of a network a recent topic of research. Understanding of human brain function has been achieved in two different ways, namely computational and data-driven approaches. Indeed, the physiological properties of living cells, in general, are determined by underlying networks of interacting genes, proteins, and neurons. These biochemical neuronal networks are extraordinarily complex and highly non-linear. To ferret out these interactions is a problem for biologists, but understanding how biochemical networks coordinate cellular responses is also a problem in applied Mathematics and Computational Biology.

The computation of the mathematical neuronal model may give clues to how neurons function smoothly in the brain. In the presence of neuronal disorders like Parkinson's disease, epileptic seizures, and even schizophrenia, mathematical models give insufficient information and in these cases a data-driven approach is more helpful. Recently, neurophysiological experiments allow collection of EEG data to identify coherent brain structures during sensory information processing. Being so relevant, it necessitates bringing further development in this subject of research with a dedicated Research Topic. This Research Topic, therefore, plans to assemble original results along with reviews on the aspects of brain function modeling at the level of neurons and their networks through computation and data-driven approaches.

The human brain connectome is one of the complicated networks that has been a challenge for researchers from different fields of science. Still, there exists a few crucial domains in which further study on mathematical modeling and neurophysiological signals *via* data-driven networks and artificial intelligence are highly needed. For instance, mathematical modeling of neuronal diseases is much less explored. Collective behaviors like synchronization and chimera states in neuronal networks are attracting a great deal of researchers' attention in recent years. The role of non-synaptic communication among neurons in order to bring chimera-like or other patterns has not been dealt with rigorously. The effect of Glial cells and astrocytes on neuronal connectivity deserves further research. The issues of information processing in complex neuronal networks and the use of artificial intelligence like machine learning or deep learning are yet to be explained in detail. Therefore, as research on brain networks study has been growing rapidly, it has become essential to advance this phenomenon with a dedicated Research Topic encircling both review works on existing studies and new original works.

In this Research Topic, Nair et al. developed a multiscale computational model to study the link between substantia nigra pars compacta (SNc) cell loss and Parkinson's disease (PD) symptoms. Their results successfully replicated the impact of SNc cell loss and levodopa (L-DOPA) medication on reaching performance. The model demonstrates how differential dopaminergic axonal degeneration in basal ganglia results in various cardinal symptoms of PD. Gao and Liu extended an influential statistical model based on the spatial classical receptive field (CRF) and non-classical receptive field (nCRF) interactions to describe the typical orientation adaptation effects observed in V1. They have shown that adaptation may link time and space by changing the “state” of the neural system according to a specific adaptor. Verma and Ambika have considered the Hindmarsh-Rose model and studied the emergence of a variety of spatio-temporal patterns among neurons that are connected in a multiplex framework, with neurons on two layers with different functional couplings. Furthermore, they discussed how the selection of these spatio-temporal patterns can be controlled by tuning the intralayer or interlayer coupling strengths or increasing the range of non-local coupling. Ambrosio and Young have shown that signals with the properties of brain rhythms can be generated from low dimensional dynamical systems such as FitzHugh-Nagumo and Leslie-Gower models. They have considered the low dimensional models with stochastically varying coefficients to replicate the qualitative characteristics of natural brain rhythms.

## Author contributions

All authors listed have made a substantial, direct, and intellectual contribution to the work and approved it for publication.

## Conflict of interest

The authors declare that the research was conducted in the absence of any commercial or financial relationships that could be construed as a potential conflict of interest.

## Publisher's note

All claims expressed in this article are solely those of the authors and do not necessarily represent those of their affiliated organizations, or those of the publisher, the editors and the reviewers. Any product that may be evaluated in this article, or claim that may be made by its manufacturer, is not guaranteed or endorsed by the publisher.

